# Identification of Key Deregulated RNA-Binding Proteins in Pancreatic Cancer by Meta-Analysis and Prediction of Their Role as Modulators of Oncogenesis

**DOI:** 10.3389/fcell.2021.713852

**Published:** 2021-11-29

**Authors:** Moumita Mukherjee, Srikanta Goswami

**Affiliations:** ^1^ National Institute of Biomedical Genomics, Kalyani, India; ^2^ Regional Centre for Biotechnology, Faridabad, India

**Keywords:** RNA-binding protein, pancreatic cancer, meta-analysis, epithelial-to-mesenchymal transition, target RNAs

## Abstract

RNA-binding proteins (RBPs) play a significant role in multiple cellular processes with their deregulations strongly associated with cancer. However, there are not adequate evidences regarding global alteration and functions of RBPs in pancreatic cancer, interrogated in a systematic manner. In this study, we have prepared an exhaustive list of RBPs from multiple sources, downloaded gene expression microarray data from a total of 241 pancreatic tumors and 124 normal pancreatic tissues, performed a meta-analysis, and obtained differentially expressed RBPs (DE-RBPs) using the Limma package of R Bioconductor. The results were validated in microarray datasets and the Cancer Genome Atlas (TCGA) RNA sequencing dataset for pancreatic adenocarcinoma (PAAD). Pathway enrichment analysis was performed using DE-RBPs, and we also constructed the protein–protein interaction (PPI) network to detect key modules and hub-RBPs. Coding and noncoding targets for top altered and hub RBPs were identified, and altered pathways modulated by these targets were also investigated. Our meta-analysis identified 45 upregulated and 15 downregulated RBPs as differentially expressed in pancreatic cancer, and pathway enrichment analysis demonstrated their important contribution in tumor development. As a result of PPI network analysis, 26 hub RBPs were detected and coding and noncoding targets for all these RBPs were categorized. Functional exploration characterized the pathways related to epithelial-to-mesenchymal transition (EMT), cell migration, and metastasis to emerge as major pathways interfered by the targets of these RBPs. Our study identified a unique meta-signature of 26 hub-RBPs to primarily modulate pancreatic tumor cell migration and metastasis in pancreatic cancer. IGF2BP3, ISG20, NIP7, PRDX1, RCC2, RUVBL1, SNRPD1, PAIP2B, and SIDT2 were found to play the most prominent role in the regulation of EMT in the process. The findings not only contribute to understand the biology of RBPs in pancreatic cancer but also to evaluate their candidature as possible therapeutic targets.

## Background

Pancreatic cancer is one of the most morbid cancer types worldwide and is mostly diagnosed at a very advanced stage with a 5-year survival rate of 8.2% (Gordon-Dseagu et al., 2018). Recent studies have identified several mutations to be predominant in different pancreatic cancer subtypes, and about 80% of these mutations are found to be sporadic (Feldmann et al., 2007; McGuigan et al., 2018). Pancreatic cancer is a heterogeneous disease with aberrant gene expression patterns like any other cancer. Therefore, in order to understand the biology of the disease, altered regulatory events need to be portrayed at the level of gene to RNA to protein to signaling pathways of the cell.

After the expression of the genes, the roadway to the functionality of the transcripts is highly regulated at the posttranscriptional and translational level. RNA-binding proteins (RBPs) are an integral part of this regulatory machinery participating in all the cellular processes. RBPs exert multi-functionality in directing the fate of the transcripts such as splicing, stability, translocation, translation, and decay (Neelamraju et al., 2015). A transcript interacts with different RBPs at different stages of life, and its fate and functionality is decided accordingly. Canonical RBPs bind to the RNAs in a sequence-specific manner with the help of RNA-binding domains (RBDs) and form dynamic ribonucleoprotein (RNP) complexes to execute the regulatory function. RBPs can bind at the coding region of the mRNA, but it is more prevalent in the regulatory elements of the RNA such as the 5′ and 3′ untranslated regions (UTRs) and in the alternative UTRs of mRNA isoforms during RNA processing (Wurth and Gebauer, 2015). There are also evidences of RBP interaction with the regulatory non-coding RNAs such as lncRNAs and miRNAs to form more complex regulatory networks. Sometimes few unconventional RBPs act as “mRNA clothes,” to expose or hide specific coding and non-coding parts of an mRNA in order to drive its activity (Singh et al., 2015; Hentze et al., 2018). Being such a pivotal player of the regulatory mechanism, the level of RBPs in a cell should be precisely regulated. So, any alterations in the expression and alteration in function due to mutation in the binding site of RBPs can break the homeostasis and lead the cell toward oncogenesis and several other diseases. There is numerous evidence of modulation of normal repertoire of RBP in a cell in several cancer types (Kechavarzi and Janga, 2014). For example, overexpression and promoter mutation of TERT are prevalent in several types of cancer such as melanoma, cholangiocarcinoma, lung squamous cell carcinoma, and adrenocortical carcinoma (Colebatch et al., 2019). ELAVL1 is a well-reported oncogenic RBP that is found to induce tumorigenesis by facilitating the translation of several growth factors and proto-oncogenes (Abdelmohsen and Gorospe, 2010; Wang et al., 2013a). Reappearance of oncofetal proteins of the IGF2BP family is a major posttranscriptional driver of oncogenesis in many aggressive cancer types (Bell et al., 2013). The RNA-binding protein FXR1 also has diverse roles in cancer progression by means of cell proliferation and viability (Jin et al., 2016; Fan et al., 2017). Ribosomal proteins control tumor suppression *via* activation of p53, and deletion (43% of tumors) and mutation in genes encoding ribosomal proteins such as RPL22 are evidenced in several cancer types such as acute lymphoblastic leukemia (Sloan et al., 2013; Pelletier et al., 2018; Lessard et al., 2019). There are evidences of some onco-ribosomes as well like RPL10-R98S mutation in T-cell leukemia mimicking the oncogenic JAK-STAT pathway (Sulima et al., 2017; Girardi et al., 2018).

Recent studies have corroborated that RBPs are highly predominant and ubiquitously expressed than any other regulatory elements throughout the cell (Wang et al., 2018). But as opposed to other cancer types, dysregulation of RBPs in the case of pancreatic cancer is not much worked out. With the recent advancement of knowledge about the multifaceted role of RBPs in cancer progression and keeping in mind the aggressive nature of pancreatic cancer, the disparity of RBPs in pancreatic cancer development needs to be explored. Here, at the beginning, we have performed a meta-analysis to identify the differentially expressed RBPs in pancreatic cancer and validated them in a separate validation dataset as well as in the Human Protein Atlas (Uhlén et al., 2015). This was followed by the identification of “hub” RBPs, finding out their coding and noncoding targets, and exploration of biological processes supposed to be altered by the deregulation of both the RBPs and their targets. Our discovery of RBPs getting involved in important oncogenic activities not only contributes to the existing knowledge but also opens up immense therapeutic possibilities.

## Methods

### Enumeration of Human RBPs

A comprehensive list of 1,542 manually curated RBPs by Gerstberger et al. (2014) was the fundamental source of RBP in our entire list of RBPs. Additionally, we downloaded a list of RBPs from the RBPDB database under the category “*Homo sapiens*” (Cook et al., 2011). Moreover, an extensive search from literature resulted in a number of RBPs (Galante et al., 2009; Kechavarzi and Janga, 2014; Neelamraju et al., 2018). We also considered RBPs from high-throughput studies involving cross-linking, mass spectrometry, etc., and a common set of proteins from these studies yielded few additional RBPs (Baltz et al., 2012; Castello et al., 2012). We finally could catalogue an exhaustive list of 1,623 RBPs (Supplementary Table S1). We obtained the Entrez IDs of these RBPs using the “org.Hs.eg.db” package of Bioconductor, and missing Entrez Ids from the package were added to the list by manual search.

### Selection of Microarray Datasets

The PDAC microarray gene expression datasets used in this study for meta-analysis were obtained from Gene Expression Omnibus (GEO) (Barrett et al., 2013). We used the keywords “pancreatic cancer”, “pancreatic adenocarcinoma,” and “pancreatic ductal adenocarcinoma” and selected only the datasets that contained information on pancreatic tumor and normal tissues of human patients. We selected five datasets for meta-analysis of the discovery cohort containing information of 241 pancreatic tumor tissues and 124 normal pancreatic tissues (Badea et al., 2008; Park et al., 2014; Janky et al., 2016; Yang et al., 2016; Chhatriya et al., 2020). Two additional datasets were chosen for meta-analysis in the validation cohort with 60 pancreatic tumor samples and 35 normal pancreatic samples (Ellsworth et al., 2013; Klett et al., 2018). Detailed information on these datasets is provided in Table 1. For validation of the RBPs in the EMT-induced pancreatic cancer cell line, a microarray gene expression dataset (GSE23952) was chosen from GEO. The dataset provides the gene expression profile of TGF-beta–induced epithelial–mesenchymal transition (EMT) in the pancreatic cancer cell line (Panc-1) compared to the normal Panc-1 cell line (Maupin et al., 2010).

**TABLE 1:** Mircoarray dataset information from GEO used for meta-analysis. The top five datasets are used for meta-analysis of the discovery cohort, and last two datasets are used for meta-analysis of the validation cohort.

Dataset type	Dataset ID	No. of tumor sample	No. of normal sample	No. of RBP probes	Platform	References
Discovery set	GSE15471	36	36	1,512	Affymetrix Human Genome U133 Plus 2.0 Array	Badea et al. (2008)
	GSE43797	7	5	1,617	Illumina HumanHT-12 V4.0 expression beadchip	Park et al. (2014)
	GSE62165	118	13	1,588	Affymetrix Human Genome U219 Array	Janky et al. (2016)
	GSE62452	69	61	1,510	Affymetrix Human Gene 1.0 ST Array	Yang et al. (2016)
	GSE143754	11	9	1,545	Affymetrix Human Transcriptome Array 2.0	Chhatriya et al. (2020)
		**241**	**124**			
Validation set	GSE16515	36	16	1,512	Affymetrix Human Genome U133 Plus 2.0 Array	Ellsworth et al. (2013)
	GSE101448	24	19	1,617	Illumina HumanHT-12 V4.0 expression beadchip	Klett et al. (2018)
		**60**	**35**			

### Meta-Analysis

Series matrix files of the datasets were downloaded from GEO, and information for only 1,623 RBPs was extracted from Log2-transformed processed data. Each dataset having the number of RBP probes is mentioned in Table 1. All the gene and probe IDs were converted to their corresponding Entrez IDs, and expression values of the same Entrez ID were aggregated with their mean value. All five microarray datasets of the discovery cohort were integrated and merged, and the corresponding combined expression set was made using the “Biobase” package of R Bioconductor. To remove batch effects among the datasets, batch correction was performed using “ComBat” function of the R Bioconductor package “sva.” Normalized data were used for the calculation of differential expression of genes in pancreatic tumor tissue compared to normal pancreatic tissue as controls. Differential expression analysis was performed using the “Limma” package of R Bioconductor (Ritchie et al., 2015). The Benjamini–Hochberg correction method was used minimize the false discovery rate (FDR). Genes with adjusted *p*-value below 0.05 and fold change (log2) cutoff of 0.5 were considered as differentially expressed genes.

### Validation

Microarray datasets GSE16515 and GSE101448 were used for validation of the DE-RBPs from the discovery cohort. Meta-analysis of these two datasets was performed as described previously, and differentially expressed RBPs below the adjusted *p*-value cutoff 0.05 were considered for validation. For validation, TCGA pancreatic adenocarcinoma (PAAD) RNA-sequencing data were also used. Differentially expressed genes from RNA-sequencing were downloaded from the GEPIA and used for validation with adjusted *p*-value cutoff of <0.01. The computation of differential gene expression from the microarray dataset of EMT-induced Panc-1 cell line (GSE23952) was performed using GEO2R (http://www.ncbi.nlm.nih.gov/geo/geo2r/). DEGs with *p*-value below 0.05 were considered as significant. The TNMplot (https://www.tnmplot.com) database was used to compare gene expression of selected RBPs in normal, tumor, and metastatic tissues (Bartha and Győrffy, 2021). Gene chip data of the pancreas from the database were taken into account for the validation. We have also used The Human Protein Atlas to obtain tissue specific expression pattern of specific RBPs. For example, for IGF2BP3, normal pancreatic image was accessed using: https://www.proteinatlas.org/ENSG00000136231-IGF2BP3/tissue/pancreas, while pancreatic cancer image was accessed using: https://www.proteinatlas.org/ENSG00000136231-IGF2BP3/pathology/pancreatic+cancer.

### Pathway Over-Representation Analysis

To explore the potential biological functions and molecular mechanisms modulated by the DE-RBPs and their coding target RNAs, we used Gene Ontology (GO) enrichment which comprises three major domains: biological process (BP), molecular function (MF), and cellular component (CC) (Ashburner et al., 2000). GO over-representation analysis was performed using the clusterProfiler package of R (version 4.0.3) with the significance threshold of *p*-value <0.05 (Yu et al., 2012). Kyoto Encyclopedia of Genes and Genomes (KEGG) is an integrated data resource for systematic analysis of gene functions (Kanehisa and Goto, 2000). KEGG pathway assignment was performed by Enrichr (https://maayanlab.cloud/Enrichr/), a web-based server for gene enrichment analysis (Kuleshov et al., 2016). Pathway enrichment for lncRNA targets of the RBPs was performed employing the LncSEA database (http://bio.liclab.net/LncSEA/index.php), a powerful platform for lncRNA enrichment analysis functions (Chen et al., 2021). DE-RBPs and their coding target RNAs were separated according to their upregulated and downregulated expression pattern in PDAC, and pathway analysis was performed separately for upregulated and downregulated groups in each category.

### Protein–Protein Interaction Network Construction and Module Screening

Validated 60 differentially expressed RBPs were mapped to the STRING database (https://string-db.org/), and the protein—protein interaction network was built with the genes of the combined score ≥0.4 (medium confidence score) (Szklarczyk et al., 2017). Then, Cytoscape software (v3.8.2) was used to visualize the interaction network (Shannon et al., 2003). The significant gene module of the network was detected by the Molecular Complex Detection (MCODE) plugin of Cytoscape with the following criteria: degree cutoff = 2, node score cutoff = 0.2, k-core = 2, and maximum depth = 100 truncation standard to comprehend the network (Bader and Hogue, 2003). Furthermore, to screen out the hub genes of the network with the highest degree of connectivity, the cytoHubba plugin of Cytoscape was used, and the top five genes from each algorithm of the analysis were together considered hub genes (Chin et al., 2014).

### Identification of RNA Targets for Selected RBPs

Targets of the RBPs, mainly those conventional RBPs that were being experimented for a long time with respect to their RNA-binding property, were derived primarily by extensive search in literature. Our next approach was to explore databases such as RNAInter (http://www.rna-society.org/rnainter/) and RNAct (https://rnact.crg.eu/), which contains information of RNAs and their interacting proteins. RNAInter (RNA Interactome Database) includes information on RNA-protein interactions based on strong and weak (high-throughput) experimental evidence and prediction algorithms (Lin et al., 2020). But, all the selected RBPs did not have experimentally validated targets and thus we also considered their targets derived from the computational prediction method. As, RNAInter does not have information for all the RBPs, we relied on the RNAct database for predicted target RNAs of the concerned RBPs (Lang et al., 2019). We also looked for target RNAs in the CLIP-sequencing database, CLIPdb (http://lulab.life.tsinghua.edu.cn/postar3/RBP.html) for protein-RNA interactions (Yang et al., 2015). Additionally, we had explored the CircInteractome database (https://circinteractome.irp.nia.nih.gov/) to investigate the interaction of RBPs with circular RNA (Dudekula et al., 2016).

## Results

### Meta-Analysis Identified Differentially Expressed RBPs in Pancreatic Cancer

Importance of the dysregulation of RBPs in cancer and the ambiguity of oncogenic RBPs as reported in different studies led us to perform a meta-analysis of multiple microarray datasets. The overall objective was to find out differentially expressed RBPs in pancreatic cancer resulting out of gene expression data from 241 tumor tissues and 124 normal pancreatic tissue samples. We initiated the process by analyzing the expression of 1,623 RBPs in these five different microarray datasets as the “discovery cohort” (Table 1), and our meta-analysis yielded a set of 49 upregulated and 21 downregulated RBPs (adj. *p*-value < 0.05, |log2FC| > 0.5). Detailed information on differential expression of these RBPs is summarized in Supplementary Table S2.

### Validation of Meta-Analysis Result

**FIGURE 1:**
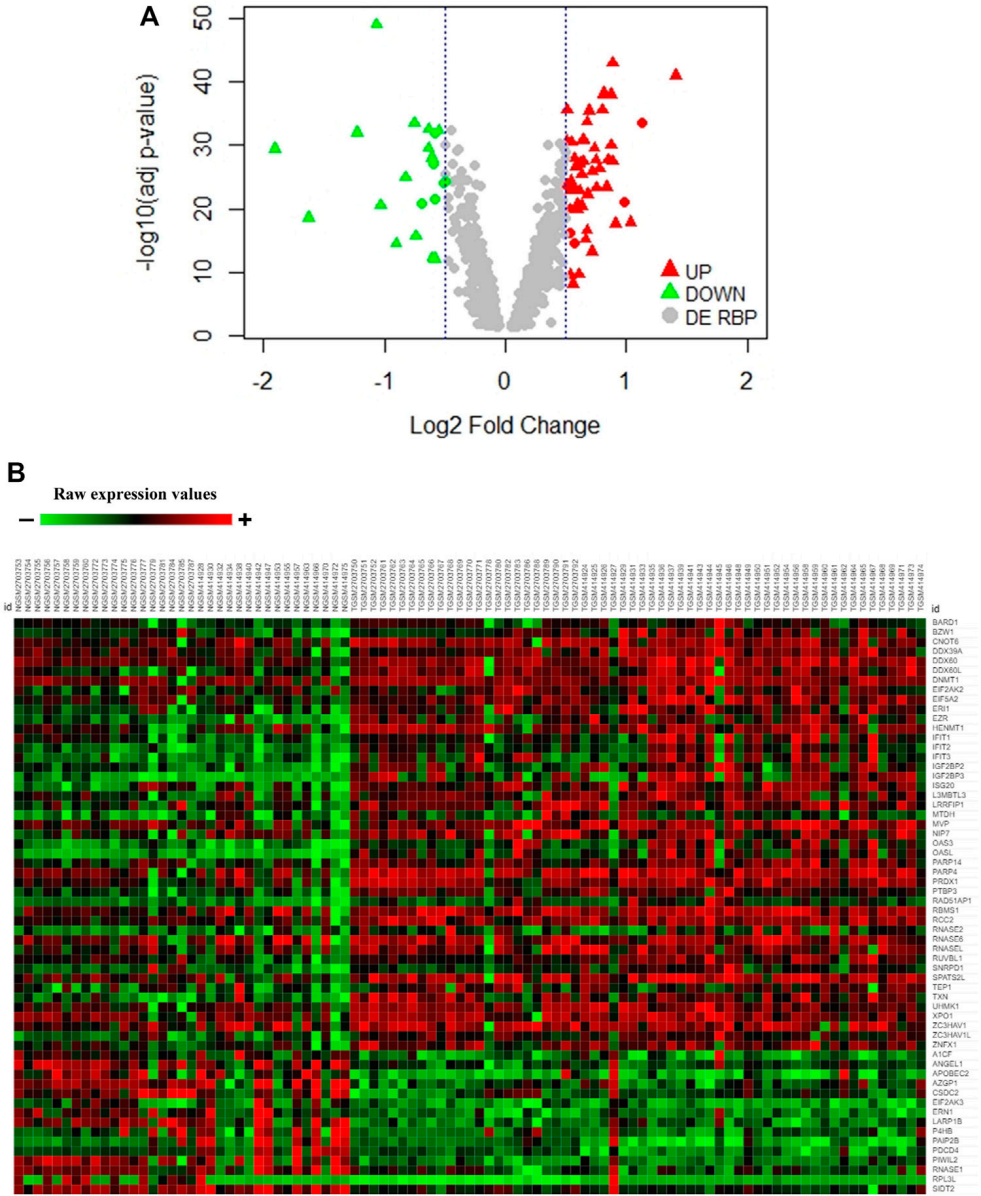
Expression pattern of DE-RBPs in pancreatic cancer. **(A)** Volcano plot showing significant DE-RBPs in the discovery dataset where red and green triangles denote validated upregulated and downregulated RBPs, respectively. **(B)** Heatmap of 60 validated DE-RBP expression in the tumor and normal tissue samples of the microarray validation dataset. Red indicates high expression and green indicates low expression.

In order to have more confidence in our findings, we further wanted to validate the differentially expressed RBPs from the discovery set separately in a validation meta-analysis set of the two microarray datasets (adj. *p*-value <0.05) (60 tumor tissues and 35 normal pancreatic tissue samples) and TCGA RNA-sequencing data of pancreatic ductal adenocarcinoma (PAAD) from GEPIA (adj. *p*-value <0.01) (179 tumor tissues and 171 normal pancreatic tissue samples) (Supplementary Figure S1). Finally, 45 upregulated and 15 downregulated RBPs in PAAD got validated following such stringent conditions (Supplementary Table S3). While the volcano plot demonstrates the distribution of validated upregulated and downregulated RBPs in Figure 1A, the heatmap portrays the expression profiles of 60 differentially expressed RBPs (45 upregulated and 15 downregulated) in the validation microarray dataset (Figure 1B). Hence, we report 60 RBPs having significantly altered expression in pancreatic cancer. Interestingly, we observed that there are reports of mutations and copy number variation in *PAIP2B*, *PRDX1*, *RNASE1*, *BARD1*, *UHMK1*, and *RPL3L* genes associated with pancreatic cancer (Bartsch et al., 2005; Barton, 2016; Tang et al., 2017; Wang et al., 2017; Alimirzaie et al., 2018; Grant et al., 2018). The finding further strengthens the importance of RBPs in pancreatic cancer.

**FIGURE 2:**
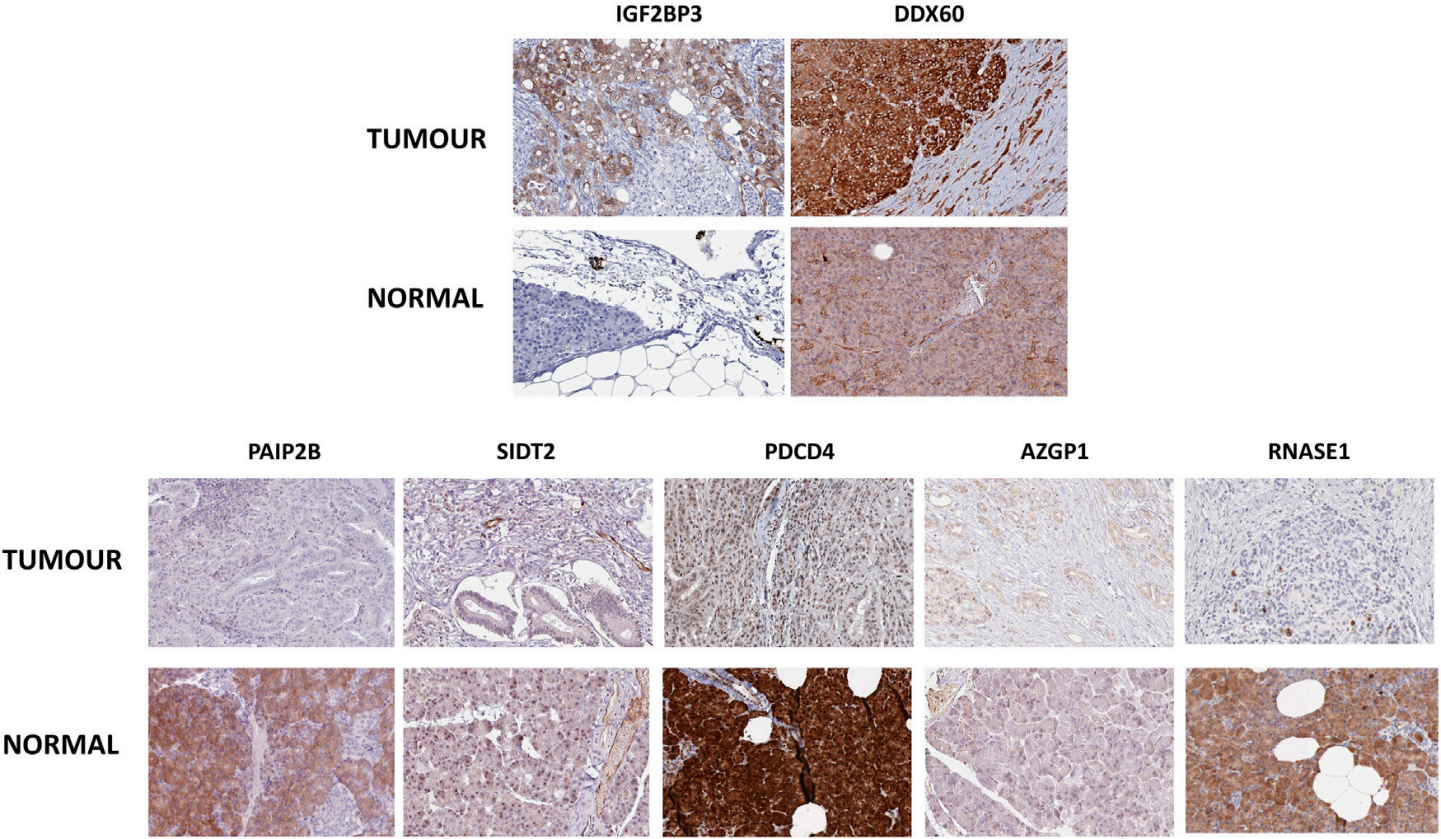
Representative Immunohistochemistry images showing expression of top-altered DE-RBPs in respective pancreatic tissues from the Human Protein Atlas Database. Upper panel shows the images of top overexpressed DE-RBPs and the lower panel shows images of most underexpressed DE-RBPs.

Next, to have an idea about the most altered RBPs, we applied a fold change cutoff of |log2FC| > 1.0 to the validated DE-RBPs identifying IGF2BP3 and DDX60 as the most upregulated and PAIP2B, AZGP1, PDCD4, SIDT2, and RNASE1 as the most downregulated DE-RBPs in PDAC. We thought that an excellent way to validate the expression of these RBPs would be to check whether these proteins were also altered in PDAC patients. Therefore, the expression of those RBPs at the protein level in pancreatic tumor tissues and normal pancreatic tissues were further investigated using immunohistochemical data from the Human Protein Atlas database. While the results for the top altered RBPs are shown in Figure 2, we found similar changes for other RBPs too. The similarity of the expression pattern of these RBPs in these patient-derived tissues to that of our transcriptome meta-analysis results further validated our finding.

### Functional Enrichment of DE-RBPs

**FIGURE 3:**
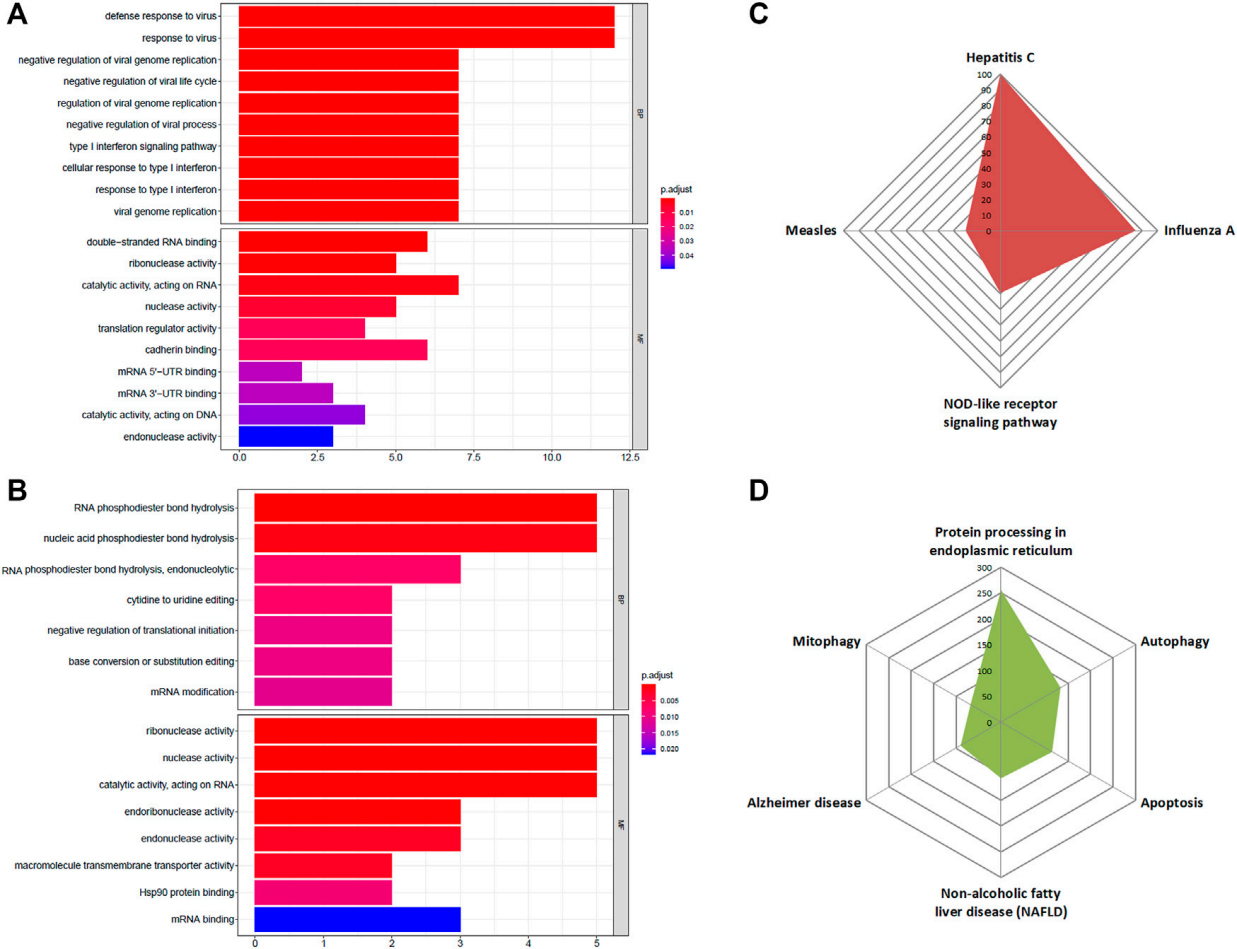
GO and KEGG enrichment analysis of DE-RBPs. **(A)** Barplot shows enriched GO terms of upregulated RBPs, **(B)** barplot shows enriched GO terms of downregulated RBPs, **(C)** radar plot represents the KEGG pathway analysis result of upregulated RBPs, **(D)** radar plot represents the KEGG pathway analysis result of downregulated RBPs. BP: biological processes, MF: molecular function.

Next, we wanted to find out what effects these altered RBPs could bring about in pancreatic cancer development and progression. To assess that, the first step would be to perform GO term enrichment and KEGG pathway analysis of DE-RBPs depicting their role in distinct biological functions and pathways. The enriched biological process (BP) section of GO displayed the immunomodulatory activity of the upregulated DE-RBPs, and the molecular function (MF) division showed that these RBPs are mainly associated with double-stranded RNA-binding, nuclease activity, translation regulation, and mRNA UTR binding (Figure 3A). On the other hand, downregulated DE-RBPs mainly exhibit phosphodiester bond hydrolysis and mRNA editing function in the BP category and stand for nuclease activity and transmembrane transport in the MF category (Figure 3B). Additionally, KEGG pathway analysis also indicated the involvement of upregulated RBPs in response to viruses such as those causing hepatitis C, influenza, and measles and in the NOD-like receptor signaling pathway which is known as a master regulator of inflammation and cancer (Saxena and Yeretssian, 2014) (Figure 3C). KEGG pathways linked to downregulated RBPs illustrate their role in protein processing, autophagy, mitophagy, and apoptosis (Figure 3D). To summarize, all the over-represented pathways either directly or indirectly substantiate the important contribution of the DE-RBPs in the pathophysiology of PDAC. The complete list of altered pathways is presented in Supplementary Table S4.

### PPI Network Construction and Identification of Significant Gene Modules

**FIGURE 4:**
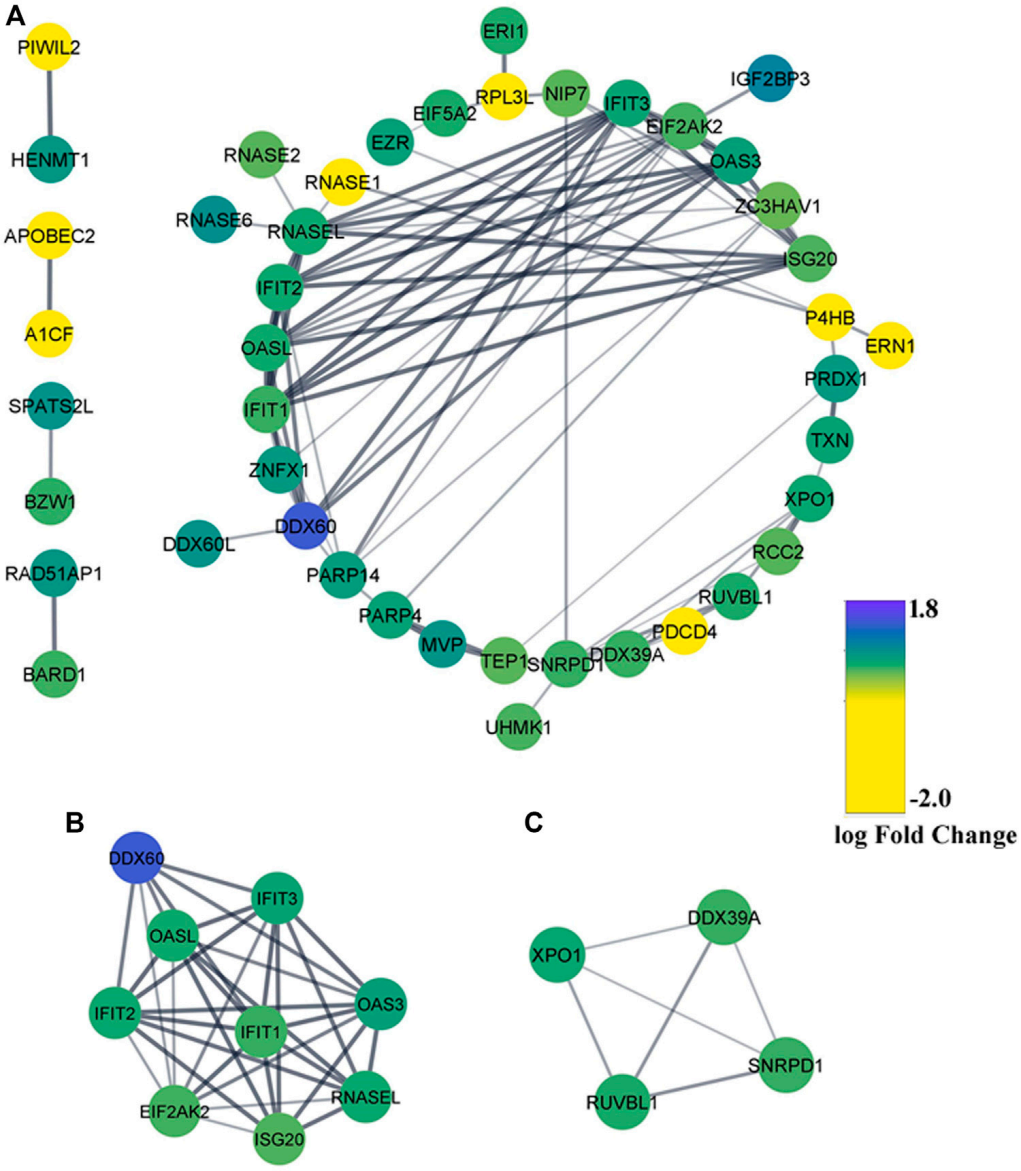
Protein–protein interaction (PPI) network of the differentially expressed RBPs. **(A)** PPI network of DE-RBPs constructed using the STRING database. RBPs without any interaction with another DE-RBP are removed. Blue denotes upregulated DE-RBPs and yellow denotes downregulated DE-RBPs. **(B,C)** Two MCODE modules from the PPI network.

Leading-edge research emphasizes higher-order interactions between RBPs to enact the combinatorial regulation of specific mRNAs and coordinate complex cellular processes (Sternburg and Karginov, 2020). To have a comprehensive insight into those intricate regulatory networks between DE-RBPs, a PPI network was formed using the STRING database and visualized with Cytoscape Software_v3.8.2. The network resulted in 44 nodes and 87 edges where blue indicated upregulated RBPs and yellow indicated downregulated RBPs (Figure 4A). The MCODE plugin was used to find the highly interconnected regions in the main network, and two significant gene cluster modules were obtained (Figures 4B,C). To recognize the important hubs or nodes in the interactome network, the cytoHubba plugin was used where hub genes were ranked by 12 topological algorithms. We used the top five genes calculated from each algorithm, and totally 20 genes were identified as the key hub genes of the network among which *EIF2AK2*, *RNASEL*, *ISG20*, *IFIT1*, and *OASL* were found to overlap in at least five methods (Supplementary Figure S2). Hence, these 20 hub genes were used in subsequent analyses with 18 genes being upregulated and 2 genes being downregulated.

### Identification of Coding and Noncoding Targets of Selected RBPs

Overall physiological functions of RBPs are governed by the type of RNA they bind with and also by the subsequent fate of those RNAs as determined by the RBPs themselves and other associated factors. MRNAs are undoubtedly the most characterized targets of RBPs, while recent discoveries of various types of noncoding RNAs and their interaction with RBPs also carry crucial functional significance. Hub genes along with the top altered DE-RBPs (|log2FC| > 1.0) (DDX60 and IGF2BP3: upregulated, PAIP2B, AZGP1, PDCD4, SIDT2, and RNASE1: downregulated) (Table 2) were taken forward for target RNA identification. The targets of these 26 RBPs; identified from literature, strong and weak experimental evidences, and bioinformatic prediction were divided into coding and noncoding categories. Noncoding RNAs were again subdivided into two categories based on their length. Long noncoding RNA groups consisted of lncRNAs and lincRNAs, pseudo-gene RNAs, circular RNAs, and few processed transcripts whereas miRNAs, snoRNAs, and scaRNAs constituted the group of small noncoding RNAs. Only the coding and long noncoding transcript targets that were differentially expressed in PDAC (GEPIA) were selected. However, the expression of small noncoding RNA targets could not be validated due to poor availability of data. A total of 203 coding targets were identified for 26 RBPs (Supplementary Table S5). We were able to identify 418 long noncoding target RNA information for 20 RBPs and small noncoding RNA information could be retrieved for 7 RBPs only (Supplementary Table S6).

**TABLE 2:** Information of the DE-RBPs used for target RNA identification. Hub RBPs along with the top-altered RBPs are included in this group. These set of RBPs are presented in the table as (A) upregulated RBPs and B) downregulated RBPs.

Up-regulated key RBPs				Down-regulated key RBPs	
A				B	
RBP	Entrez ID	RBP	Entrez ID	RBP	Entrez ID
DDX60	5549161	EIF2AK2	5549161	AZGP1	5549161
IFIT1	5568	IFIT2	5568	P4HB	5568
IFIT3	258882	IGF2BP3	258882	PAIP2B	258882
ISG20	88855	MVP	88855	PDCD4	88855
NIP7	2225	OAS3	2225	RNASE1	2225
OASL	51235	PARP4	51235	SIDT2	51235
PRDX1	91615	RCC2	91615		
RNASEL	99161	RUVBL1	99161		
SNRPD1	575527	TEP1	575527		
ZC3HAV1	33683	ZNFX1	33683		

### Target RNAs Drive Key Oncogenic Pathways

**FIGURE 5:**
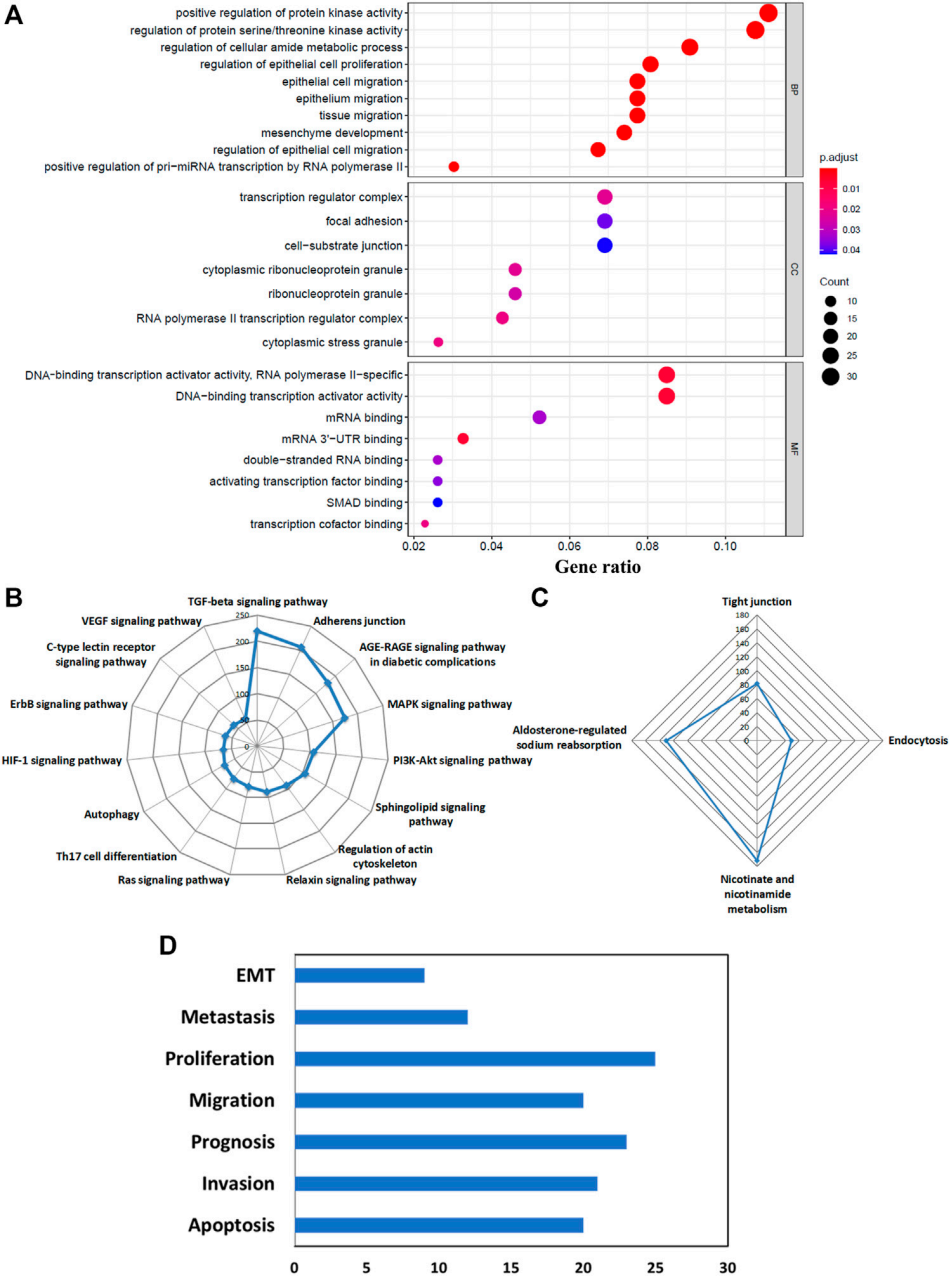
Biological functions and pathways of the predicted target RNAs of DE-RBPs. **(A)** Bubble plot shows the enriched GO terms of upregulated coding targets: biological processes (BP), Cellular Component (CC), and molecular functions (MF). **(B)** Radar plot shows KEGG pathway analysis of upregulated coding targets. **(C)** Radar plot is shows KEGG pathway analysis of downregulated coding targets. **(D)** Altered pathways represent cancer hallmarks for predicted lncRNA targets from the LncSEA database.

As mentioned before, the function of an RBP depends on the target RNAs it interacts with and hence, the pathways or biological processes modulated by these targets largely determine the role of the RBP in general; both in normal physiological conditions as well as in the diseased state. Therefore, it is imperative to explore the functions of both the coding and noncoding targets identified for our selected RBPs. Upregulated coding RNA targets underwent GO term enrichment and KEGG pathway analysis. The biological process (BP) segment of GO depicted the role of the target RNAs mainly in the regulation of epithelial cell proliferation and migration and mesenchymal development which indicated their involvement toward epithelial-to-mesenchymal transition (EMT). The regulation of protein kinase and serine/threonine kinase emerges as the top biological process mediated by the RBP targets, which is also a salient regulator of EMT. Positive regulation of pri-miRNA transcription by the RBP targets is also a significant biological process which denotes the direct and indirect involvement of the RBPs in miRNA biogenesis along with their targets (Figure5A). The GO-enriched terms of the cellular component exhibited the association of the target RNAs with transcription regulator complex, focal adhesion, cell-substrate junction, ribonucleoprotein granule, and cytoplasmic stress granules (Figure 5A). In the molecular function (MF) category, coding targets are mainly enriched in DNA-binding transcription activator activity, transcription cofactor binding, mRNA, double-stranded RNA, and UTR binding (Figure 5A). As the number of downregulated coding RNA targets is very less, we did not get any significant enriched GO terms for those. The KEGG pathway analysis of upregulated coding targets delineated their role in several signaling pathways such as the TGF-beta signaling, AGE-RAGE signaling, MAPK signaling, PI3K-Akt signaling, sphingolipid signaling, relaxin signaling, Ras signaling, HIF-1 signaling, ErbB signaling, C-type lectin receptor signaling, and VEGF signaling pathway. Additionally, they are also indicated to be involved in adherens junction, regulation of actin cytoskeleton, and Th17 cell differentiation (Figure 5B). Downregulated KEGG pathways due to downregulated coding genes involve nicotinate and nicotinamide metabolism, aldosterone-regulated sodium absorption, tight junction, and endocytosis (Figure 5C). In brief, we observed the alteration of very important pathways known to be involved in cancer development and progression, through the modulation of coding targets by RBPs. The list of all altered pathways is shown in Supplementary Table S7.

Like coding targets, we also wanted to find out the downstream pathway modulation by the long noncoding RNA targets of RBPs. The long noncoding Set Enrichment Analysis (LncSEA) webtool was used for this purpose and to our surprise, pathways leading to cancer cell migration, metastasis, and EMT were the most important ones (Figure 5D).

### Epithelial-to-Mesenchymal Transition Emerged as the Most Prevalent Biological Process From RBP Targets

**FIGURE 6:**
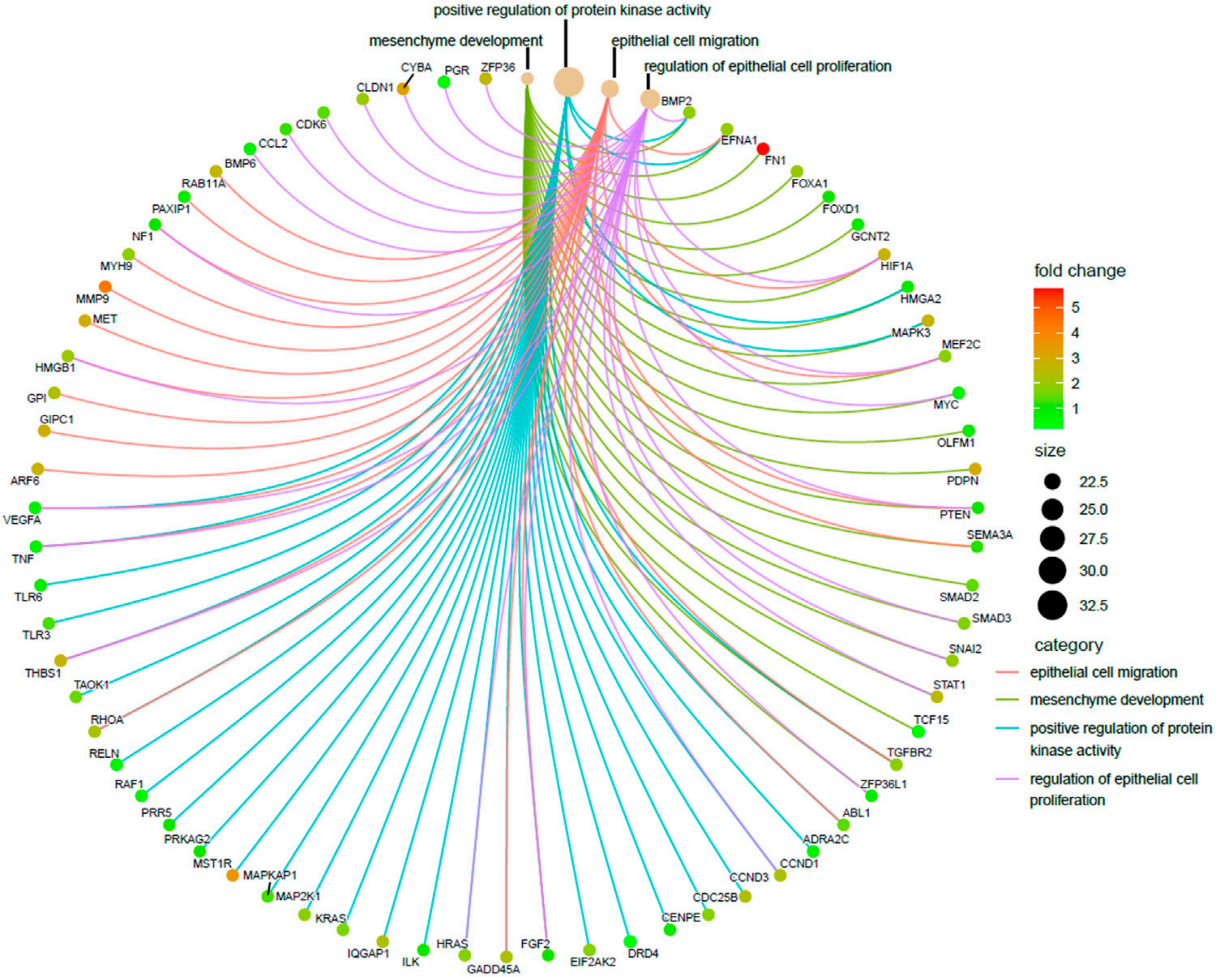
Visualization of enriched EMT-related GO terms of upregulated coding targets. Cnetplot showing four EMT-related enriched GO terms of the biological process (BP) category derived from upregulated coding targets along with the associated targets. Red represents the upregulated targets and green represents the downregulated targets.

As already described in the previous segment, epithelial-to-mesenchymal transition (EMT) turned out to be the most distinct pathway modulated by the predicted targets of the selected RBPs. EMT is a process by which epithelial cells lose their cell polarity and cell–cell adhesion and attain migratory and invasive properties becoming the cell type of mesenchymal characteristics, and the process is considered a crucial step for tumor metastasis. We were curious to know exactly what were the RBPs which play a central role in this process and what were their targets. In order to do so, four EMT-related GO-enriched terms of the biological process (BP) category were selected from upregulated coding target–driven pathways, as shown in Figure 6 and a cnetplot was used to visualize the responsible target genes. The next obvious step was to go back and identify the RBPs targeting those RNAs and actually driving the process. Interestingly, we found that these were the target genes of all 26 RBPs used in the analysis. The result tells us that there is a clear indication that deregulated RBPs primarily promote EMT and thereby facilitate metastasis in pancreatic cancer.

**FIGURE 7:**
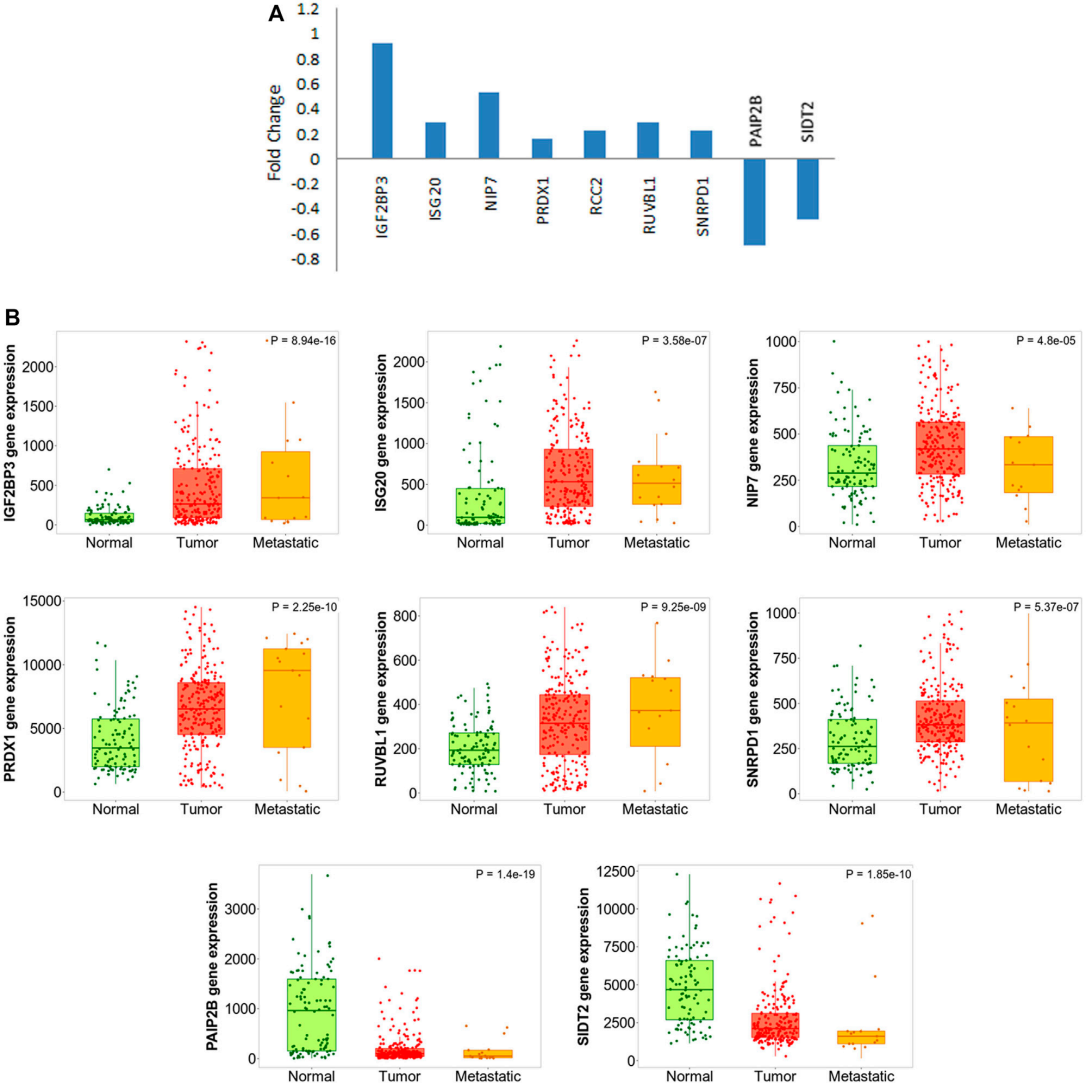
DE-RBP expression deregulated in EMT and metastasis. **(A)** Barplot shows the expression pattern (log2 fold change) of DE-RBPs to be differentially expressed in TGF-β–induced EMT in the Panc-1 cell line, as compared to uninduced Panc-1 cells, as obtained from the database GSE23952. **(B)** Boxplot comparing the expression of the abovementioned DE-RBPs in normal, tumor, and metastatic tissue samples as computed using the TNMplot database.

Considering the fact that the finding is quite significant with respect to pancreatic carcinogenesis, we went on further to validate the results. There have been several experiments where the effect of any particular EMT-promoting factor has been tested on cancer cell lines by monitoring gene expression status at both epithelial and mesenchymal states. We found that one dataset (GSE23952) where TGF-β was used to induce EMT in Panc-1 pancreatic cancer cells, and gene expression profiling was performed before and after induction. We understand that the treatment by TGF-β cannot be the only method to induce EMT and might also not completely mimic the actual scenario prevailing in PDAC patients. Still, we moved forward and used this dataset to validate the expression of our selected RBPs over there, keeping in mind that the TGF-β–induced EMT model is the most widely used method, and the dataset is the best available option at present. We could validate the expression of nine RBPs in that dataset (Figure 7A), thereby supporting their role in EMT and indicating that TGF-β could also play a possible role here.

In another attempt to further validate our finding, we wanted to check what is the pattern of the expression of these RBPs during metastatic progression of the disease in actual patient data. If an RBP is either a promoter or inhibitor of EMT, we can expect that it is progressively up or downregulated expression from the normal pancreatic tissue to primary tumor to metastatic lesion. We used a web-platform TNMplot which is an integrated database using transcriptome-level datasets to compare gene expression in normal, tumor, and metastatic tissues. Expression values of these EMT-related RBPs were checked. Progressive nature of the expression of the RBPs which already got validated in TGF-β–mediated EMT induction study further validated their importance in EMT (Figure 7B). Additionally, the expression of remaining 19 RBPs was also tested using TNMplot, and 12 out of 18 RBPs were progressively altered, strengthening our finding that these RBPs play a significant role in EMT in pancreatic cancer (Supplementary Figure S3). Expression information on RCC2 and ZNFX1 were not found in the dataset.

Lastly, in order to further verify the expression status of altered RBPs in pancreatic cancer cell lines and tissue samples, we explored the published literature and used The Protein Atlas data. There were multiple studies where actually the expression of these specific RBPs was estimated in several pancreatic cancer cell lines or patient tissues using qPCR and/or Western blotting or immunohistochemical analysis; convincingly supporting our results (Supplementary Table S8) (Fernández-Salas et al., 2000; Kong et al., 2010; Bhatti et al., 2011; Taniuchi et al., 2014a; Cai et al., 2015; Taniuchi et al., 2015; Schultz et al., 2017; Tang et al., 2017; Yu et al., 2017; Nguyen et al., 2019; Cui et al., 2020; Konishi et al., 2021). Immunohistochemistry results for IGF2BP3, PAIP2B, and SIDT2 have already been shown in Figure 2, and we additionally present the results for the rest of the five RBPs in pancreatic cancer tissues as shown in Supplementary Figure S4, which strongly validate their expression in patient samples signifying their importance in the disease.

## Discussion

The prime objective of this study was to identify the deregulated RBPs and investigate the modulation of gene expression by them in pancreatic cancer. As RBPs play an instrumental role in regulating normal cellular processes, the altered expression of RBP emerged as a major player in the development and progression of cancer. Apart from candidate RBP studies and their involvement in pancreatic cancer, there are very few reports of universal RBPome studies derived from TCGA RNA-sequencing data in pancreatic cancer which have discrepancies between the results (Glass et al., 2020; Wen et al., 2020). Disparity among the outcomes of these studies led us to investigate the RBPs which are universally altered in pancreatic cancer. The best unbiased approach we thought of was to access all available relevant gene expression microarray datasets and process them together to find out the expression information for the RBPs and perform the analysis.

The meta-analysis offers statistical methods to integrate results from multiple comparable studies with the aim of extrapolating the number of observations and statistical power and provide a precise estimate of the effects of an intervention (Fagard et al., 1996). Thus, we conducted a meta-analysis of five published transcriptomic microarray datasets for the discovery cohort, and the differentially expressed RBPs were validated by another meta-analysis of two more transcriptomic array datasets and DE-RBPs from TCGA RNA-sequencing data. Eventually, we identified 45 overexpressed and 15 underexpressed DE-RBPs that were invariably claimed to be differentially expressed in pancreatic cancer (Figure 1). Minute observations to the set of altered RBPs reflected the enrichment of some protein families such as IGF2BP, APOBEC, and OAS along with the assembly of DEAD-box helicases, RNAse, translation initiator, vault proteins, and some interferon-induced proteins. There are also some unconventional RBPs with no functional validation of their RNA-binding activity such as SPATS2L, TXN, UHMK1, ANGEL1, and P4HB. RBPs associated with innate immune response and immunomodulation are immensely represented in DE-RBPs, as also shown by the pathway enrichment. IFIT, OAS, and ZC3HAV family proteins are distinct interferon-stimulated genes (ISGs) (Justesen et al., 2000; Brice et al., 2019; Pidugu et al., 2019) whereas LRRFIP1 and PDCD4 are involved in the regulation of type I interferon response (Yang et al., 2010; Kroczynska et al., 2012). Another interesting feature is the downregulation of negative regulators of the typical translational machinery (EIF2AK3 and PDCD4) to combat the high energy demanding process of general translation (Supplementary Table S3). Altered expression of translation mediators indicate toward “translation acrobatics,” that is, exploiting alternate modes of translation initiation by cancer cells (Sriram et al., 2018). For example, the non‐canonical translation initiation factor EIF2AK2 is found to be upregulated, and the upregulated EIF5 determines the frequency of the leaky scanning mode of alternative translation and is reported as a novel oncogenic protein (Wang et al., 2013b). DDX60 was revealed to be the most overexpressed RBP in pancreatic adenocarcinoma. DDX60 is an ATP-dependent RNA helicase which induces type I IFN (IFN-α/β), and IFN- β is reported to influence oncogenic Ras transformation (Miyashita et al., 2011; Tsai et al., 2011). But there is no report of DDX60 involvement in pancreatic cancer, in spite of its consistent upregulation. IGF2BP3 is another important overexpressed KH-domain–containing RBP which is already reported to promote invasiveness and metastatic properties of the PDAC cells (Taniuchi et al., 2014b). Most downregulated RBPs include PAIP2B, SIDT2, PDCD4, AZGP1, and RNASE1, among which we report for the first-time involvement of PAIP2B, SIDT2, PDCD4, and RNASE1 in pancreatic cancer (Figure 2).

Some DE-RBPs exhibit protein–protein interaction among themselves, and the interaction network regulates several cellular processes. We also tried to find out the hub RBPs while searching for the RBPs having more connectivity and thus more regulatory capacity (Figure 4). RBPs act as the principal mediators of posttranscriptional regulation of gene expression by directly targeting various types of RNAs. Many experimental methods have been employed to identify the potential RNA partners of a particular RBP. Deep-sequencing approaches are the most convenient method nowadays to uncover the RBP-RNA interactome, but strong bioinformatic prediction methods can also help us identify targets of novel RBPs, which can serve as the basis for further prospective studies. However, we have tried to look for the targets of a definite RBP in every possible way to estimate the functions and pathways governed by the target RNAs of altered RBPs. Evidence from pathway enrichment intuitively manifested the connection between downstream target RNAs and EMT, a major event in metastasis (Figure 5). Being a highly dynamic process, metastatic cascade is also known to be promoted by the differentiation of tumor-infiltrating immune cells. Immune evasion of the cancer cells at the primary site of the tumor remotely prepares their pre-metastatic niche (Kitamura et al., 2015). Given the prevalence of immune response–related terms in RBP annotation results, as mentioned in Figure 3, it is worth justifying the potential role of target RNAs in pancreatic cancer metastasis. Apart from the direct involvement of target RNAs in promoting metastasis or EMT, concerned RBPs also act as direct or indirect regulators of EMT. We validated the expression of RBPs in a gene expression dataset of the TGF-β–induced EMT cell line of pancreatic cancer (Figure 7). There we came across nine RBPs differentially expressed in an identical pattern of our DE-RBPs. As the mode of regulation by RBP is a complex process, the expression level of target RNAs is not always linear with the expression of that particular RBP. Hence, the direction of altered expression is not always predictable using this approach. Moreover, TGF- β is not the sole inducer of EMT, and in the tumor microenvironment, there are other paracrine signaling pathways such as WNT pathway, NOTCH signaling, and mitogenic growth factor such as EGF and FGF-mediated activation pathways acting in combination to induce an EMT program (Dongre and Weinberg, 2019). Each pathway triggers EMT by modulating different sets of RBPs. As we have chosen a cell-line based model of EMT induced by a particular signaling pathway, the system primarily lacks other signaling modules and cross-talk with infiltrating immune cells which are known to be major player of EMT. Thus, the RBPs we found altered in TGF-β–induced EMT might be specific for the process. However, it does not mean that other RBPs do not have any contribution in EMT as they could always be part of other signaling pathways promoting EMT. The TNMplot dataset lastly verified the relatedness of the selected RBPs with EMT by comparing between normal, tumor, and metastatic tissue samples (Figure 7). We felt that we have tried to validate our findings by multiple means. Even, at the end, we had curated the published reports of experimental observations of other multiple groups where they had evaluated the expression of these RBPs in pancreatic cancer cell lines and patient samples (Supplementary Table S8). Still, detailed experimental interrogation of the functional role of individual RBPs in cell lines or animal models could have the final say on establishing their mechanism of action during the regulation of EMT.

Therefore, our study provides a comprehensive view of the dysregulated RBPs in pancreatic cancer and substantial insights into the molecular mechanisms of how RBPs interact among themselves and with their target RNAs, modulating one of the important hallmarks in pancreatic cancer, the epithelial-to-mesenchymal transition. “RBP”-eutics is a currently emerging field where RBPs are gaining attention as a promising target for cancer therapeutics using diverse approaches such as small-molecule inhibitors and oligonucleotide-based strategy (Mohibi et al., 2019). Thus, considering the importance of our identified RBPs in pancreatic cancer, their candidature as a suitable drug target will be worth investigating.

.

## Data Availability

The original contributions presented in the study are included in the article/Supplementary Material; further inquiries can be directed to the corresponding author.

## Supplementary Material

Supplementary Figure S1 Flowchart for analyzing DE-RBPs in pancreatic cancer. Discovery cohort includes meta-analysis of five microarray datasets, and DE-RBPs from the analysis were validated in a meta-analysis to two microarray datasets and TCGA RNA-sequencing data.

Supplementary Figure S2 Highest degree connectivity network of the top five hub genes for each topological algorithm. Top 5 hub genes for each 12 topological algorithms are identified from the cytoHubba plugin used in Cytoscape. Interaction networks are based on their highest degree of connectivity. Each network denotes each algorithm used: **(A)** Betweenness, **(B)** BottleNeck, **(C)** Closeness, **(D)** Clustering coefficient, **(E)** Degree, **(F)** Density of Maximum Neighborhood Component (DMNC), (G) EcCenticity, **(H)** Edge Percolated Component (EPC), **(I)** Maximal Clique Centrality (MCC), **(J)** Maximum Neighborhood Component (MNC), **(K)** Radiality, and **(L)** Stress.

Supplementary Figure S3 Boxplot showing the role of hub RBPs and top-altered RBPs in metastasis. Boxplot compares the expression of the hub RBPs and top-altered RBPs in normal, tumor, and metastatic tissue samples from the TNMplot database. Expression of the remaining six RBPs is shown in Figure 7A.

Supplementary Figure S4 Immunohistochemistry images showing the expression of EMT-specific RBPs in respective pancreatic tissues from the Human Protein Atlas database. Comparison of the expression of tumor and normal tissue samples has been shown. The database does not have any IHC staining data corresponding to ISG20.

Supplementary Table S1 List of enumerated RBPs. The list includes the names of 1623 RBPs along with their Entrez IDs.

Supplementary Table S2 Differentially expressed RBPs in pancreatic cancer from the meta- analysis of the discovery cohort. The table includes analyzed data of the RBPs in pancreatic tumor tissues compared to normal pancreatic tissues identified from the meta-analysis of the five microarray datasets.

Supplementary Table S3 Validated differentially expressed RBPs in pancreatic cancer. DE- RBPs from the discovery cohort are validated in a meta-analysis of the two microarray datasets and TCGA RNA-sequencing data. The validated DE-RBPs which include 45 upregulated and 15 downregulated RBPs are listed in the table.

Supplementary Table S4 GO term enrichment and KEGG Pathway analysis results of DE- RBPs. Result of GO enrichment for upregulated RBPs is shown in sheet 1 named as “UP RBP GO” and result for downregulated RBPs shown in sheet 2 named as “DOWN RBP GO.”Sheet 3 named as “KEGG” includes the result of KEGG pathway analysis for upregulated and downregulated RBPs.

Supplementary Table S5 Interacting coding RNA partners of the selected DE-RBPs.

Supplementary Table S6 Interacting noncoding RNA partners of the selected DE-RBPs. Noncoding target RNAs are divided into two categories depending on their size. First sheet of the file consists of the “Long Noncoding RNA” targets of the RBPs. This category includes the name of RBPs, name of interacting RNA, and biotype of the target transcripts such as lncRNAs and lincRNAs, pseudo-gene RNAs, circular RNAs, and few processed transcripts. Second sheet of the file consists of the “Small Noncoding RNA” targets of the RBPs. This category includes the name of RBPs, name of interacting RNA, and biotype of the target transcripts such as miRNAs, snoRNAs, and scaRNA.

Supplementary Table S7 GO term enrichment and KEGG pathway analysis results of coding targets of selected RBPs. Result of GO enrichment for upregulated coding targets are shown in sheet 1 named as “UP TARGET GO.” KEGG pathway analysis results of upregulated coding targets are shown in sheet 2 named as “UP TARGET KEGG”, and results of downregulated coding targets are shown in sheet 3 named as “DOWN TARGET KEGG.”

Supplementary Table S8 List of published reports of experimental validation verifying the expression status of altered RBPs in pancreatic cancer cell lines and patient samples.

## Glossary

RBP: RNA-binding protein
TERT: telomerase reverse transcriptase
ELAVL1: ELAV-like RNA-binding protein 1
FXR1: FMR1 autosomal homolog 1
JAK-STAT: Janus kinase- signal transducer and activator of transcription
TCGA: The Cancer Genome Atlas
GEPIA: gene expression profiling interactive analysis
DDX60: DEAD-box helicase 60
IFN: Interferon: Interferon
IGF2BP: Insulin-like growth factor 2 mRNA-binding protein
PDAC: Pancreatic ductal adenocarcinoma
PAIP2B: Polyadenylate-binding protein–interacting protein 2B
SIDT2: SID1 transmembrane family member 2
PDCD4: Programmed cell death 4
AZGP1: Alpha-2-glycoprotein 1
RNASE1: Ribonuclease A family member 1
NOD: Nucleotide-binding oligomerization domain
ISG20: Interferon-stimulated exonuclease gene 20
MAPK: Mitogen-activated protein kinase
AGE: Advanced glycation end products
RAGE: Receptor of AGE
PI3K-Akt: phosphatidylinositol 3-kinase - AKT serine/threonine kinase
HIF: Hypoxia-inducible factor
VEGF: Vascular endothelial growth factor
Th17: T-helper cell 17
RCC2: Regulator of chromosome condensation 2
APOBEC: Apolipoprotein B MRNA editing enzyme catalytic subunit
OAS: Oligoadenylate synthetase
SPATS2L: Spermatogenesis-associated serine rich 2–like
TXN: Thioredoxin
UHMK1: U2AF homology motif kinase 1
ANGEL1: Angel homolog 1
P4HB: Prolyl 4-hydroxylase subunit beta
IFIT: Interferon-inducedpProtein With tetratricopeptide repeats
LRRFIP1: LRR-binding FLII-interacting protein 1
EIF2AK: Eukaryotic translation initiation factor 2 alpha kinase
EIF5: Eukaryotic translation initiation factor 5
IFN: Interferon: Interferon
TGF- β: Tumor growth factor-beta
lncRNA: Long noncoding RNA
WNT: Wingless-type
EGF: Epidermal growth factor
FGF: Fibroblast growth factor
SnoRNA: Small nucleolar RNA
ScaRNA: small Cajal body-specific RNA
IHC: Immunohistochemistry
