# Supplementary figures and images for: Identification of Key Deregulated RNA-Binding Proteins in Pancreatic Cancer by Meta-Analysis and Prediction of Their Role as Modulators of Oncogenesis
Source: Front Cell Dev Biol. 2021 Nov 29;9:713852. doi: 10.3389/fcell.2021.713852 (PMC8667787; doi:10.3389/fcell.2021.713852)

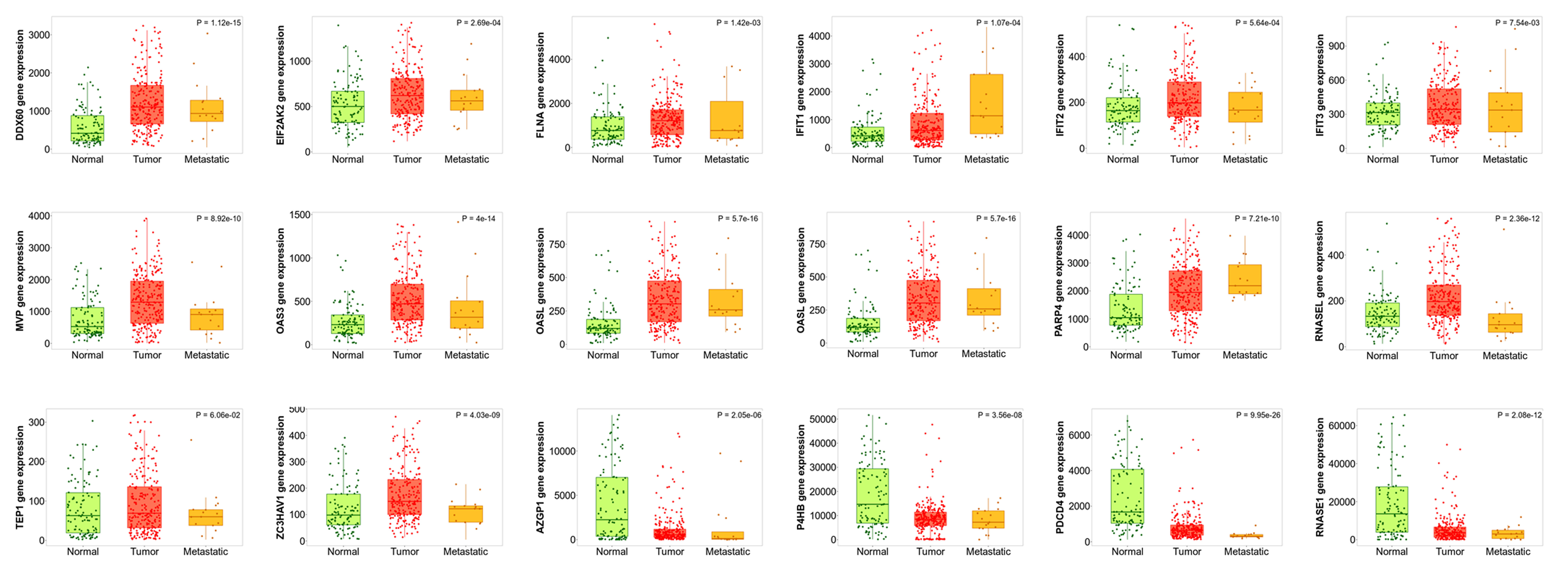

Supplement: Supplementary file 2 [file Image3.JPEG]

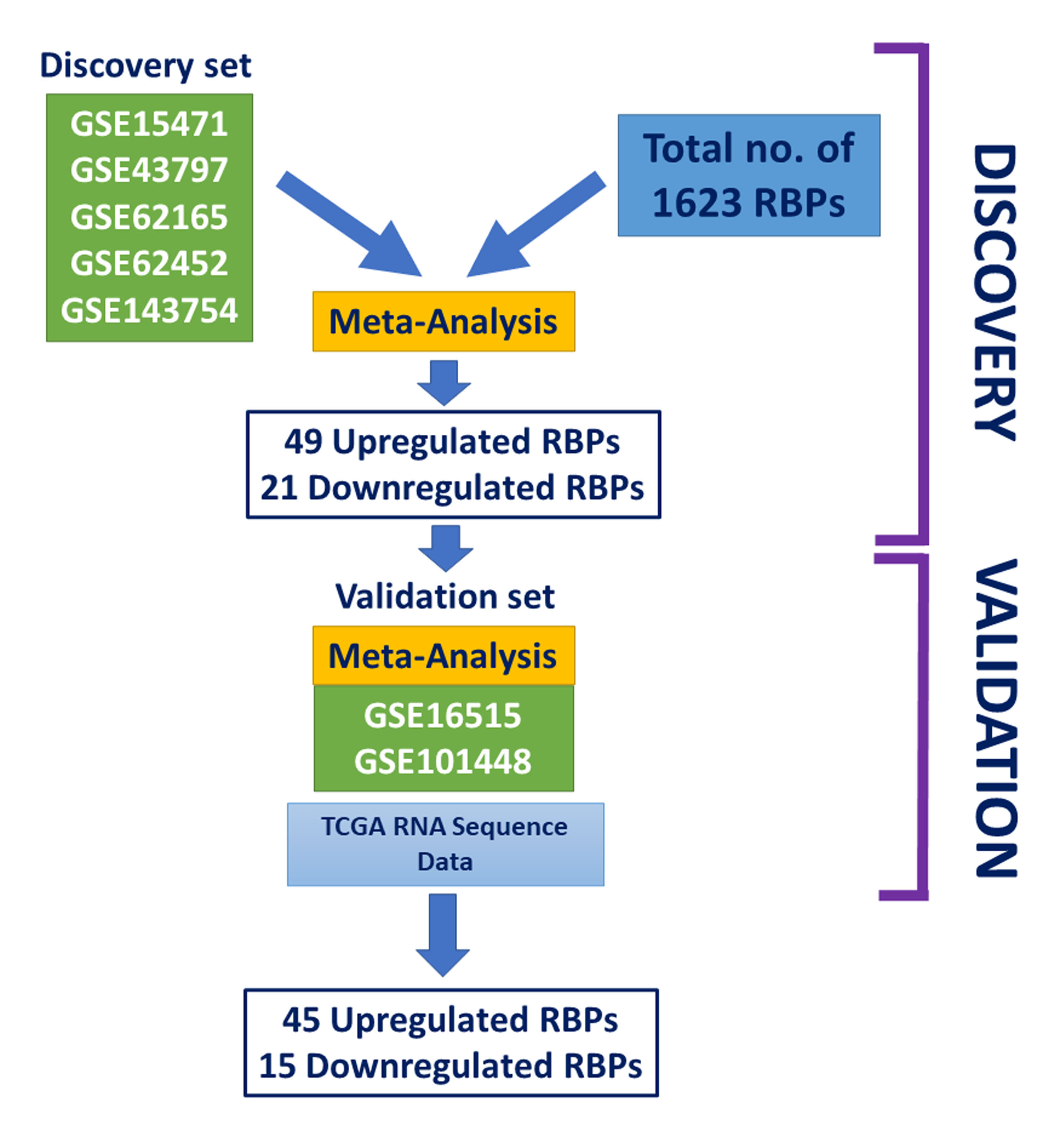

Supplement: Supplementary file 5 [file Image1.JPEG]

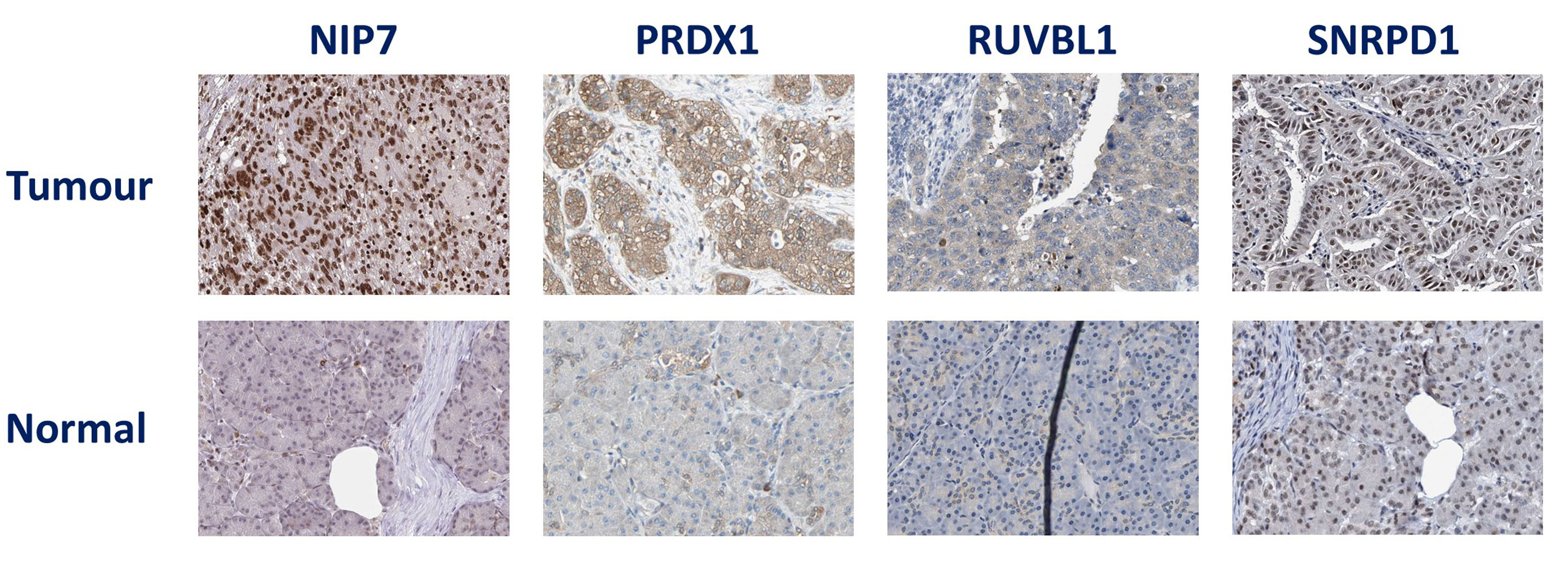

Supplement: Supplementary file 6 [file Image4.JPEG]

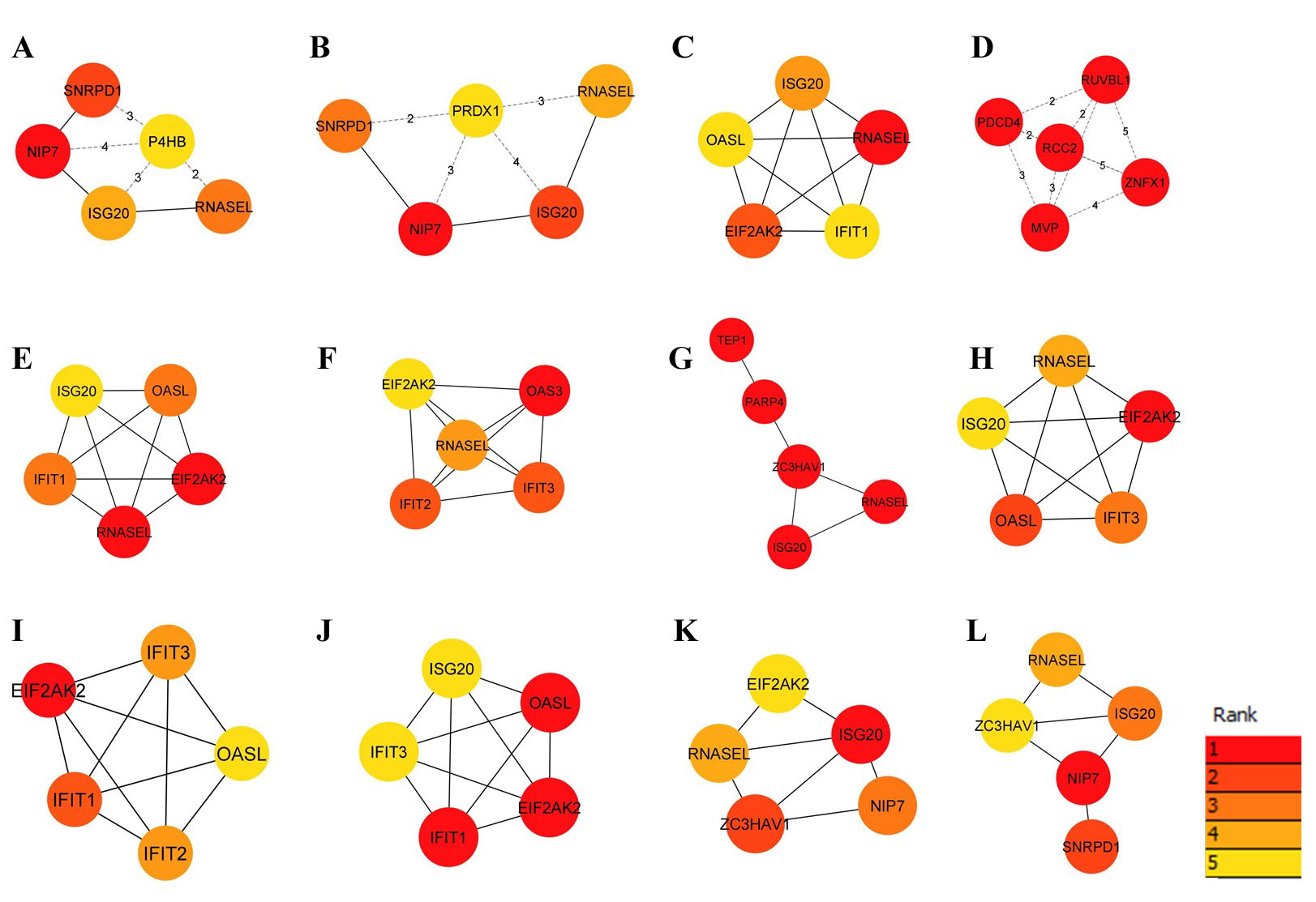

Supplement: Supplementary file 7 [file Image2.JPEG]
