# Supplementary material for: Identification of Key Deregulated RNA-Binding Proteins in Pancreatic Cancer by Meta-Analysis and Prediction of Their Role as Modulators of Oncogenesis
Source: Front Cell Dev Biol. 2021 Nov 29;9:713852. doi: 10.3389/fcell.2021.713852 (PMC8667787; doi:10.3389/fcell.2021.713852)
Supplement: Supplementary file 4 [file Table8.DOCX]

**Supplementary Table 8**

| **RBP Name** | **Expression Status** | **Pancreatic Cancer Cell Line**  **and/ or**  **Patient Tumour Samples** | **Method of Validation** | **Reference (PMID)** | **Remarks** |
| --- | --- | --- | --- | --- | --- |
| IGF2BP3 | Upregulated | cell lines:  ASPC-1  SW1990  PANC-1  MIA Paca-2 | qPCR  Western blot | 33246429 | **High expression detected in Pancreatic Cancer cell lines by qPCR and Western Blot** |
| PRDX1 | Upregulated | human PDAC tissue sections from 5 patients | IHC | 25426613 | **High expression detected in Pancreatic Cancer patient tissues by IHC** |
|  | Upregulated | Human PDAC tumor tissue  cell lines:  Panc1  ASPC1 | qPCR  Western blot  IHC | 29190947 | **High expression detected in Pancreatic Cancer cell lines and patient tissue samples by qPCR and Western Blot** |
|  | Upregulated | Pancreatic cancer tissues | IHC | 25434328 | **High expression detected in Pancreatic Cancer patient tissues by IHC** |
| RUVBL1 | Upregulated | cell lines:  S2-013  Panc1 | Western blot | 24728183 | **High expression detected in Pancreatic Cancer cell lines by Western Blot** |
| SNRPD1 | Detected | cell lines:  Panc1  SUIT-2 | Functional Analysis | 34202873 | **Expression detected in Pancreatic Cancer cell lines** |
| PAIP2B | Downregulated | Pancreatic cancer tissues | High throughput  RNA-seq. and subsequent validation | 28470677 | **Reduced expression detected in Pancreatic Cancer patient tissues** |
| SIDT2 | Downregulated | Pancreatic cancer tissues | High throughput  RNA-seq. | 31546103 | **Reduced expression detected in Pancreatic Cancer patient tissues** |
| PDCD4 | Downregulated | cell lines:  MIA PaCa-2 | qPCR | 21088996 | **Reduced expression detected in Pancreatic Cancer cell lines by qPCR** |
|  | Downregulated | cell lines:  SW1990 | qPCR  Western blot | 29218103 | **Reduced expression detected in Pancreatic Cancer cell lines by qPCR and Western Blot** |
| AZGP1 | Downregulated | cell lines:  AsPC-1  Capan-1  Human PDAC tumor tissue | qPCR  Western blot | 20581862 | **Reduced expression detected in Pancreatic Cancer cell lines and patient tissue samples by qPCR and Western Blot** |
| RNASE1 | Detected | cell lines:  AsPC-1  Capan-1  MDAPanc-3 | Northern Blot | 10691987 | **Expression detected in Pancreatic Cancer cell lines by Northern Blot** |
